# Microglial CD300f immune receptor contributes to the maintenance of neuron viability in vitro and after a penetrating brain injury

**DOI:** 10.1038/s41598-023-43840-1

**Published:** 2023-10-05

**Authors:** Daniela Alí-Ruiz, Nathalia Vitureira, Hugo Peluffo

**Affiliations:** 1 Neuroinflammation and Gene Therapy Lab., Institut Pasteur de Montevideo, Montevideo, Uruguay; 2 Departamento de Histología y Embriología, Facultad de Medicina, UdelaR, Montevideo, Uruguay; 3 Departamento de Fisiología, Facultad de Medicina, Universidad de la República, Montevideo, Uruguay; 4 Unitat de Bioquímica i Biología Molecular, Departamento de Biomedicina, Facultat de Medicina i Ciències de la Salut, Universitat de Barcelona (UB), Barcelona, Spain; 5 Institut de Neurociències, Universitat de Barcelona (UB), Barcelona, Spain

**Keywords:** Inflammation, Innate immune cells, Innate immunity, Neuroimmunology, Cell death in the nervous system, Cellular neuroscience, Glial biology, Neuroimmunology

## Abstract

Emerging evidences suggest that immune receptors participate in diverse microglial and macrophage functions by regulating their immunometabolism, inflammatory phenotype and phagocytosis. CD300f, a TREM2-like lipid sensing immune receptor, that integrates activating and inhibitory cell-signalling pathways, modulates inflammation, efferocytosis and microglial metabolic fitness. In particular, CD300f overexpression was described to be neuroprotective after an acute brain injury, suggesting a role for this immune receptor in neurotrophic interactions. Thus, we hypothesised that CD300f modulates neuronal survival through neuron-microglial interactions. In order to study its biological function, we used in vitro and in vivo approaches, CD300f^−/−^ animals and rCD300f-Fc, a fusion protein that interrupts the endogen interaction between CD300f receptor-ligands. In hippocampal cocultures containing neurons and mixed glia, we observed that rCD300f-Fc, but not control IgGs induced neuronal death. In accordance, in vivo studies performed by injecting rCD300f-Fc or control IgGs into rat or WT or CD300 KO mice neocortex, showed an increased lesioned area after a penetrating brain injury. Interestingly, this neuronal death was dependent on glia, and the neurotoxic mechanism did not involve the increase of proinflammatory cytokines, the participation of NMDA receptors or ATP release. However, exogenous addition of glial cell line-derived neurotrophic factor (GDNF) prevented this process. Taken together, our results suggest that CD300f modulates neuronal survival in vitro and after a penetrating brain injury in vivo and that CD300f inhibition alters microglial phenotype generating a neurotoxic microenvironment.

## Introduction

Immune receptors play a critical role in regulating immune and inflammatory processes in the central nervous system (CNS). They primarily function by adjusting the threshold and duration of myeloid cell responses^1,2^. The CD300 family of immune receptors consist of several activating and inhibitory members^2^. The existence of a CD300-like molecule in ancient vertebrates and the maintenance of its function predicts an essential role of this family in innate immunity^3,4^. On the other hand, CD300f has a particular interest given its ability to mediate activating and inhibitory signals in myeloid innate immune cells^5,6^ and its modulatory role in microglial immunometabolic phenotype and synaptic pruning^7^. In line with its role in modulating inflammatory phenotypes, it has been shown that CD300f mediates anti-inflammatory signaling by decreasing mast cell degranulation and allergic responses^8^ and counteracting macrophage activation by interfering with several TLR signaling cascades^9^. On the contrary, the antibody-dependent activation of CD300f in microglia cultures potentiates lipopolysaccharide (LPS)/TLR4 mediated responses^10^. Moreover, CD300f ligands are present in the CNS in vivo and in nervous system primary cultures^11^, and CD300f overexpression after an acute brain injury is neuroprotective^11^. However, the role of CD300f signaling in cell–cell neurotrophic interactions is not well understood.

The CD300f immune receptor shares many characteristics with TREM2, a key immune receptor for determining the phenotype of microglia and macrophages, and one of the main risk factor genes for the development of Alzheimer’s Disease (AD) and Nasu Hakola Disease^1^. In accordance, mounting evidence showed that CD300f has been associated with a neuroprotective response against Tau pathology^12^ further highlighting its pivotal role in nervous system function in both health and disease^7,13^. Interestingly, CD300f is among the most up-regulated genes in brain microglia/macrophages after several proinflammatory insults such as intraperitoneal LPS injection^7,14^, spinal cord injury^15^, or with age in a mouse tauopathy model^12^. Moreover, its upregulation has been associated to promyelinating microglia after a demyelinating stimuli^16^. The inhibition of CD300f using a soluble rCD300f-Fc fusion protein delayed peripheral nerve regeneration after a sciatic nerve injury^17^. Genetic variants of CD300f have also been associated to non-classical inflammatory functions of microglial and potentially CNS barrier-associated macrophages (BAM), leading to the modulation of neuropsychiatric conditions such as major depressive disorder and anxiety^7,18^. In spite of all these accumulated functional data indicating a role for CD300f and its ligands in neuronal survival, the underlying cellular mechanisms are not well understood.

To explore the role of CD300f in neuroprotection, we used CD300f deficient mice (CD300f^−/−^) and the rCD300f-Fc fusion protein which interrupts the endogenous interaction between CD300f receptor and its ligands, both in vitro and in vivo^17^. By using hippocampal cocultures containing neurons and glia, we observed that CD300f inhibition promotes neuronal death compared to controls. This effect was dependent on glia, because no cell death was detected in enriched hippocampal neuronal cultures treated with rCD300f-Fc. Moreover, we found that conditioned media derived from rCD300f-Fc-treated cocultures induced neuronal death when applied to enriched neuronal cultures. However, conditioned media derived from enriched glial cultures treated with rCD300f-Fc did not affect neuronal survival. In accordance with these results*, *in vivo studies performed by injecting rCD300f-Fc or control IgGs into wild type (WT) rat or mouse neocortex showed an increased lesioned area. However, no effect on the lesion volume was observed after injecting rCD300f-Fc in CD300f^−/−^ mice, suggesting that the effect of the rCD300f-Fc fusion protein is specific. Taken together, our data suggest that CD300f plays a key role in neuroprotection and that neuron-glia interactions are essential in this process, probably by promoting the release of gliotransmitters. Moreover, CD300f inhibition may alter microglial phenotype generating a neurotoxic microenvironment.

## Methods

### Cell cultures

Animal care and protocols were approved by the Committee of Ethics in Animal Research (CHEA-UDELAR), Uruguay. Dissociated hippocampal neuron-glia cocultures were prepared from P0–P1 rat pups as described previously with minor modifications^19^. Coverslips were coated by adding a 5 mM acetic acid solution containing 5 μg/ml poly-d-lysine (Sigma-Aldrich) and 0.4 mg/ml rat-tail collagen. Mixed glial cultures were plated with BME-based culture media (GibCo) containing 10% fetal calf serum (GibCo), Glutamax (GibCo), 20 mM d-glucose (GibCo), HEPES (1%), pyruvate (1%) and penicillin/streptomycin (GibCo). After 6–7 days in vitro (DIV), dissociated hippocampal cells were plated onto a glial monolayer and maintained in Neurobasal-based culture media (GibCO) containing B-27 (GibCo), Glutamax, 20 mM d-glucose and penicillin/streptomycin. After 24–48 h AraC (4 μm, Sigma-Aldrich) was added to minimize proliferation. Cultures were used for experiments at 14–15 DIV^20^. Conditioned media were generated by treating mixed glial cultures or cocultures at 12 DIV for 72 h with rat CD300f-Fc (1.0 μg/ml, Sinobiological custom bulk produced without preservatives) or control IgG (1.0 μg/ml). Enriched hippocampal neuronal cultures were prepared by seeding 150,000 neurons in 12 mm coverslips, which were manually counted by using a Neubauer counting chamber with trypan blue to exclude dead cells. After 12 DIV, they were treated for 72 h with rCD300f-Fc or control IgG, or with conditioned media diluted 1:2 alone or with GDNF; 1.0 ng/ml), CPP (3-(2-Carboxypiperazin-4-yl)propyl-1-phosphonic acid, a selective N-methyl-d-aspartate (NMDA)-type receptor antagonist; 100 μM), or CBX (carbenoxolone, the gap junction locker; 100 μM).

### Immunocytochemistry

Cells were fixed in 4% paraformaldehyde (PFA 4%, Sigma-Aldrich), 15 min at room temperature (RT), permeabilized with PBS containing 0.1% TritonX-100 (PBS-T, Sigma-Aldrich), and blocked in PBS (Sigma-Aldrich) containing 0.2 M glycine, 10% FBS (Sigma-Aldrich), and 0.1% TritonX-100 (Sigma-Aldrich), for 1h at RT. For estimating cell numbers, the following primary antibodies were added in PBS containing 5% FBS and incubated for 2h at RT: mouse anti-βIII Tubulin (1:1000, Cat. No. G7121, Promega, USA), mouse anti-APC (1:300, Cat. No. OP80, Calbiochem, Germany), rabbit anti-IBA1 (1:1000, Cat. No. 019-19741, Wako, Japan), or rabbit anti-GFAP (1:1000, Cat. No. Z0334, DAKO, Denmark). After three washes with PBS-T cells were incubated with secondary fluorescently conjugated antibodies Alexa Fluor 488 or 594 (Invitrogen) and DyLight 649 (Jackson ImmuneResearch). Negative controls, without primary antibody incubation, were made to rule out non-specific staining. DAPI (1:300, Sigma, Milwaukee, Wisconsin, USA) was used for nuclei detection. As an assay for evaluating the specificity of rCD300f-Fc neurotoxicity, the following primary antibodies were used: mouse anti-mouse myeloid-associated immunoglobulin-like receptor five (MAIRV)/CMRF-35-like molecule1 (CLM1) (1:20, MAB27881 R&D Systems, Minneapolis, MN, USA) and goat anti-mouse CLM1/CD300f (1:50 Invitrogen, Catalog # PA5-47,399).

### MTT assay

Cell viability was indirectly estimated by the MTT assay, which is based on the cellular reduction of tetrazolium salts. Cells were incubated with MTT (final concentration 0.5 mg/ml) for 2 h for allowing intracellular reduction of the soluble MTT to the insoluble formazan dye. Then, the media was carefully removed without disturbing the formazan crystals formed by viable cells and 100 µL of dimethyl sulfoxide (DMSO) was added to solubilize the formazan crystals. Absorbance was measured at 570 nm using a microplate reader (Thermo/Labsystem Multiskan MS, Thermo Fisher Scientific, Waltham, MA USA).

### Image analysis

Images were acquired on an epifluorescence microscope (Olympus IX8) at 20X and analyzed using Fiji software. All pictures were obtained following the same pattern by generating a cross and placing its center at the center of the well. One picture was taken per field, and 15 fields in total were used per well. The number of βIII-Tubulin-positive neurons per well was determined by summing neurons from all photos acquired per well. In each experiment, the number of βIII-Tubulin-positive cells were counted and normalized to control conditions (untreated cells).

### *Animals and *in vivo* injection*

All experimental work was approved by the UDELAR Ethical Commission (Exp. No. 070153-000528-14) and the Ethics Commission for the Use of Animals (CEUA) of the Institut Pasteur de Montevideo (No. 006-20) and conducted according to directives of the Federation of Laboratory Animal Science Associations (FELASA). Data are reported in accordance with ARRIVE guidelines. The animals used in this study were: adult (8–10 weeks) 6.Cg-Tg(Thy1-YFPH)2Jrs male mouse heterozygote for yellow fluorescent protein (YFP-H) (Jackson Laboratories), adult (4 to 5 months old) male C57BL/6 wild type (WT) mice and CD300f^−/−^ mice (Genentech, Bar Harbor, ME, USA^13^), adult (4 to 5 months old) male Wistar rats. Animals were injected at neocortex (coordinates L: − 0.15; V: − 0.1 cm) using a stereotaxic frame under isoflurane (Abbott, Abbott Park, IL, USA) anesthesia and a nanoinjector (2 μl at 0.4 μl/min during 5 min; Quintessential Stereotaxic Nanoinjector, Stoelting CO. Wood Dale, IL, USA) with saline solution (0.9% NaCl), rCD300f-IgG (30 μg/ml) or control IgG (30 μg/ml). The needle was left in place for an additional 5 min to allow diffusion into the brain parenchyma. Treatments were randomly distributed among animals in each cage. Three days after the lesion was performed, animals were anesthetized and perfused intracardially with 4% PFA in 0.1 M phosphate buffer (pH 7.4). Brains were post-fixed with PFA 4% (2 h), cryoprotected in 30% sucrose, and frozen with CO_2_. Parallel cryostat coronal Sects. (30 μm) of the entire brain were prepared, stained with Nissl and used for quantification of the lesioned and total area of the hemisphere. The brain tissue sections were immersed in toluidine blue solution (15 ml of solution A composed by 0.5% toluidine blue in water and 200 ml of solution B composed by three parts of acetic acid 0.2 M and two parts of sodium acetate 0.2 M) for 20 min, rinsed with distilled water, dehydrated in ethanol solutions with increasing concentrations and cleared in xylene. Then, the sections were mounted with DPX mounting medium (Sigma-Aldrich), covered with cover slips, and observed under a light microscope. Damaged neurons were identified by loss of Nissl staining, whereas healthy neurons exhibited Nissl substance within the cytoplasm, a more relaxed chromatin structure, and well-defined nucleoli.

Quantification of the lesioned Nissl pale area was performed using Fiji software with parallel microscope observation (using 10X and 20X magnification). The lesion area and the total lesioned hemisphere area were used to calculate the lesion volume and the total (ipsilateral) hemisphere volume; data were expressed as “% of lesioned hemisphere” to correct for the possible edema effect. Neurotoxicity in YFP mouse brains was determined by the number of YFP-positive neurons counted in the contralateral hemisphere minus those counted in the injured hemisphere. Values were expressed as “% of neuronal death”.

### Statistical analysis

One-way analysis of variance (ANOVA) followed by Holm-Sidak´s test or Tukey’s post-hoc analysis were used for experimental data with normal distribution. Statistical analyses were performed with Prism 8 software and data was shown as mean ± SEM, considering *p* < 0.05 as statistically significant. The number of independent experiments performed are indicated in the figures. We did not perform any outlier test and did not eliminate any potential outlier.

### Ethical approval

All experimental work was approved by the UDELAR University Ethical Commission (Exp. No. 070153-000528-14) and the CEUA if the Institut Pasteur de Montevideo (No. 006-20) conducted according to directives of the Federation of Laboratory Animal Science Associations (FELASA).

## Results

### CD300f inhibition in neuron-glial cocultures is neurotoxic

**Figure 1:**
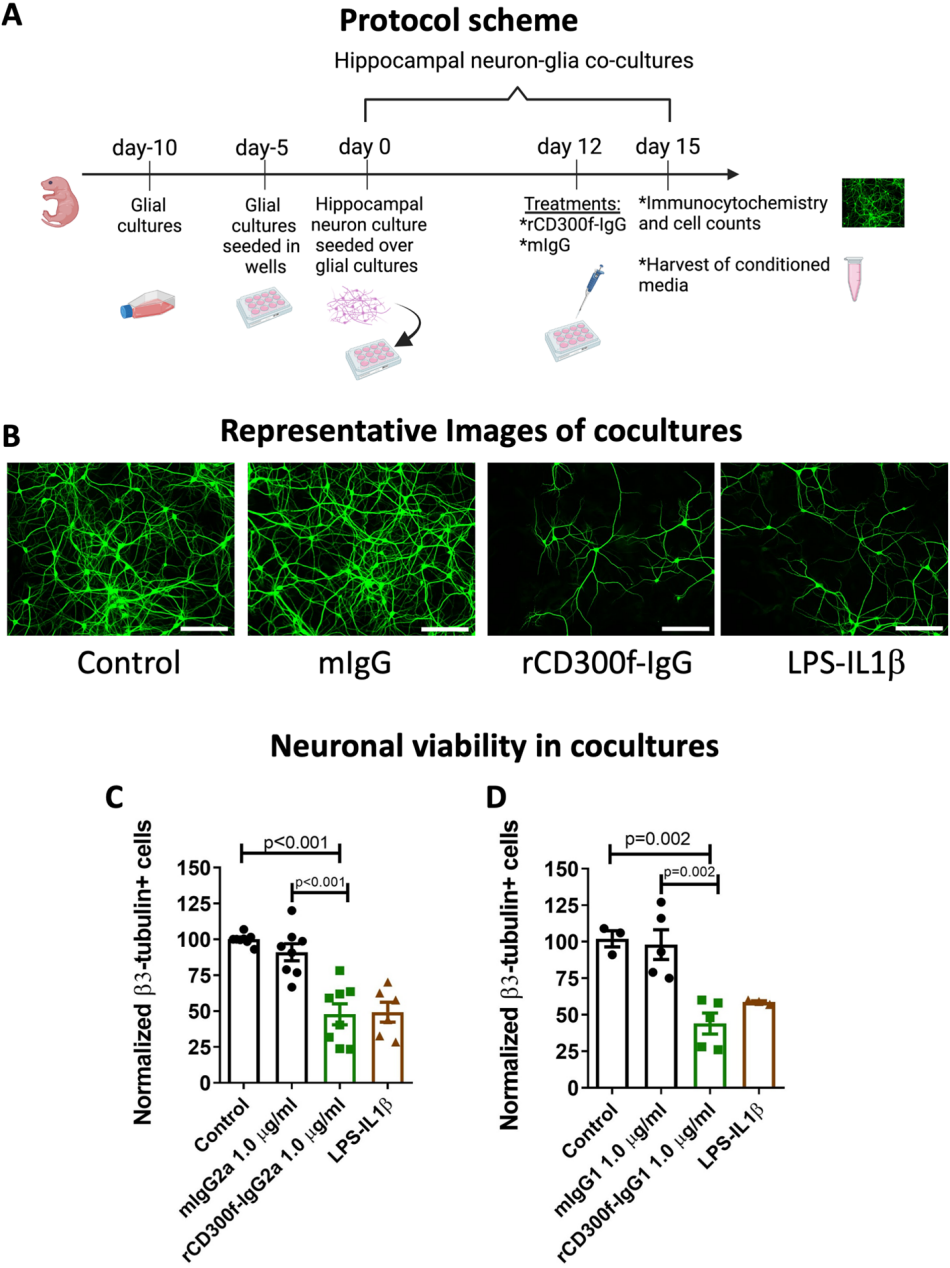
CD300f inhibition induced neurotoxicity in vitro. (**A**) Protocol scheme. (**B**) Representative images of neurons immunolabeled with β3–tubulin antibody in untreated cocultures, and treated with: mIgG2a, rCD300f-IgG, or LPS + IL1β. Scale bars 400 μM. (**C**,**D**) β3–tubulin-positive cells were counted 72 h post-incubation. Values are expressed as normalized to untreated controls. Four independent experiments were performed in (**C**) and (**D**) (two for each IgG isotype). Data show mean ± SEM; p corresponds to one way ANOVA followed by Tukey’s test.

In order to explore the role of CD300f in modulating neuron-glia interactions underlying neuroprotection in vitro, we first analyzed the cellular composition of hippocampal dissociated cocultures by immunolabeling against several cell-type specific markers. We found 36% βIII-Tubulin positive neurons, 38% GFAP positive astrocytes, 11% IBA1 positive microglia and 15% APC positive oligodendrocytes (Suppl. Figure 1). Next, we analyzed neuronal survival 72h after treating cocultures (at 12 DIV) with the soluble fusion protein rCD300f-Fc (1.0 μg/ml) or with control IgGs (1.0 μg/ml) (Fig. 1A,B). The rCD300f-Fc protein is composed by the extracellular domain of rat CD300f fused to a mouse IgG2a Fc region (rCD300f-IgG2a). This fusion protein inhibits the activation of CD300f by its endogenous ligands being a widely used strategy for modulating immune receptors^1,17^. Interestingly, treating cocultures with rCD300f-IgG2a induced a 52 ± 17% and 43 ± 17% decrease in the number of βIII-Tubulin positive neurons when compared to the untreated or mIgG2a controls, respectively (Fig. 1B,C). Moreover, incubation with LPS (1.0 μg/ml, 72h) together with IL1β (10 ng/ml, 72h), a well-known neurotoxic proinflammatory insult, showed a similar decrease in the number of βIII-Tubulin-positive neurons (51 ± 17%) (Fig. 1B,C). These data suggest that rCD300f plays a pivotal role in neuronal survival.

The IgG2a isotype can activate complement and Antibody-Dependent Cellular Cytotoxicity (ADCC). Thus, to avoid the putative non-specific effects of the rCD300f-IgG2a fusion protein, we generated and additional fusion protein composed by the extracellular domain of rat CD300f fused to the IgG1 Fc region, which has lower complement activation capacities and decreased ADCC triggering^21^. The incubation of hippocampal cocultures with rCD300f-IgG1 fusion protein (1.0 μg/ml for 72h) mimics the effect of CD300f inhibition by rCD300f-IgG2a, showing 60 ± 16% and 55 ± 16% of neuronal cell death when compared to untreated or mIgG2a controls, respectively (Fig. 1D). To further confirm the specificity of the neurotoxic effect, hippocampal cocultures were treated with rCD300f-IgG1 protein preincubated with two different blocking polyclonal anti-CD300f antibodies (rat anti-CD300f (R&D Systems) and goat anti-CD300f (Invitrogen). Notably, the neurotoxic effect was fully abolished in both cases when compared to their respective specific IgG controls or the anti-CD300f antibodies alone (Suppl. Figure 2).

### CD300f inhibition in vivo is neurotoxic after a penetrating brain injury

**Figure 2:**
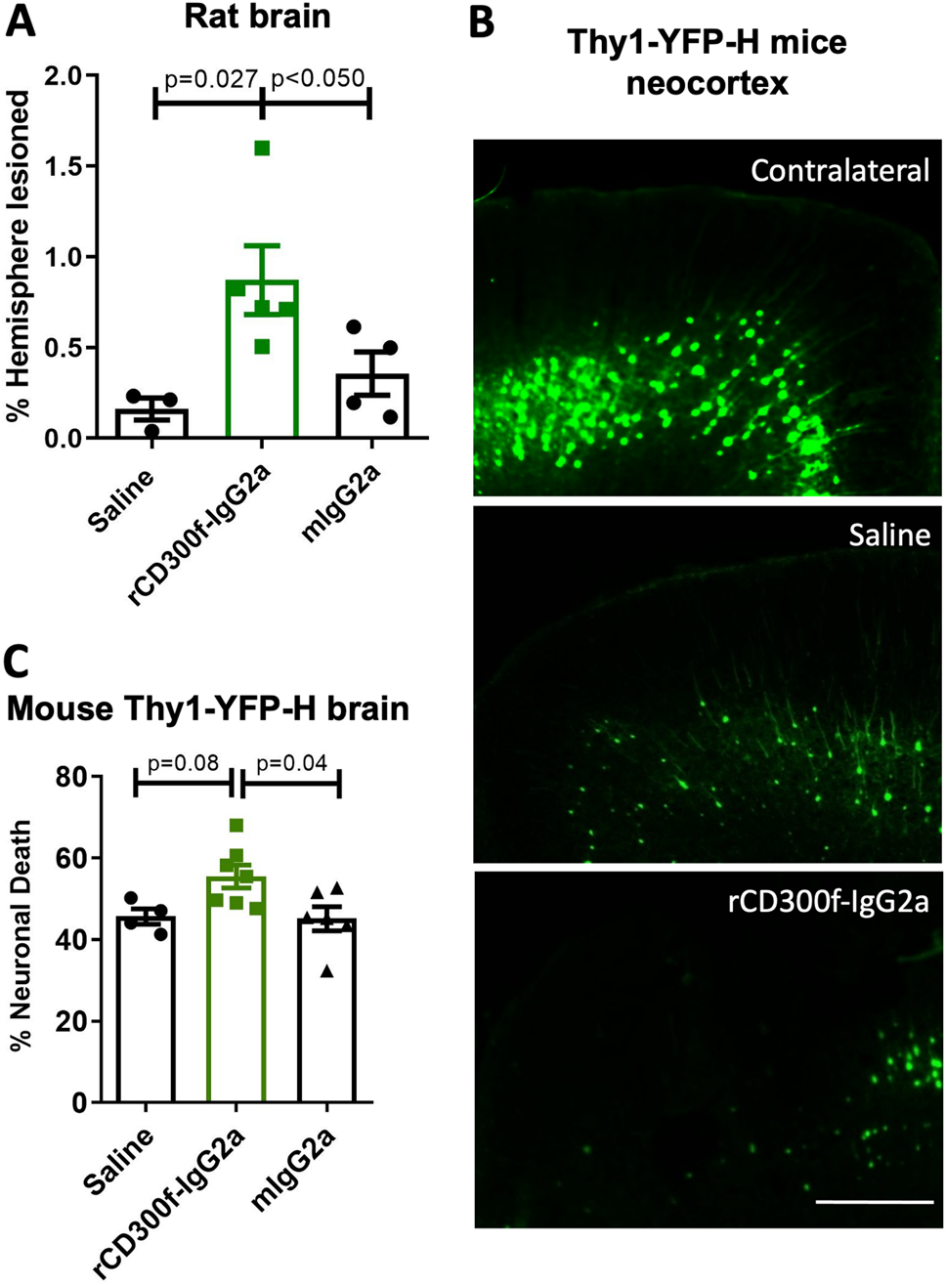
CD300f inhibition induced neurotoxicity in vivo. (**A**) Bar graphs showing the percentage of lesion volume in rat cortex 72 h after being injected with saline (n = 3), rCD300f-IgG2a (n = 5, 30 μg/ml), or control IgG2a (n = 4, 30 μg/ml) and measured by Nissl staining. (**B**) Representative images of mouse Thy1-YFP-H cortex injected with saline or rCD300f-IgG2a and contralateral non-injected cortex (Scale bars 100 μM). (**C**) Bar graph showing the number of YFP + cortical neurons in Thy1-YFP-H cortex after intracortical injection of saline (n = 4), rCD300f-IgG2a (n = 7, 30 μg/ml), or control IgG2a (n = 6, 30 μg/ml) Data show mean ± SEM; p corresponds to one way ANOVA followed by Holm-Sidak´s test.

To explore the role of CD300f in modulating neuronal survival in vivo, we injected saline, rCD300f-IgG2a (30 μg/ml) or control IgG2a (30 μg/ml) into rat neocortex and evaluated the lesion volume by Nissl staining (Fig. 2A). In accordance with our in vitro results, inhibiting CD300f after a penetrating cortical injury increase the % of lesioned hemisphere compared to controls injected with saline or mIgG2a (saline 0.16 ± 0.1%, rCD300f-IgG2a 0.87 ± 0.42% and mIgG2a 0.36 ± 0.24%) (Fig. 2A), suggesting that CD300f promotes neuronal survival both in vivo and in vitro. An increased neuronal death was also observed after neocortical injection of rCD300f-IgG2a (30 μg/ml) in Thy1-YFP-H mice, compared to saline or control IgG2a (30 μg/ml) (saline 46.2 ± 4.5%, rCD300f-IgG2a 58.2 ± 6.7% and mIgG2a 48.3 ± 4.6%)(Fig. 2B,C). Together these results suggest that blocking microglial CD300f signaling in vivo reduces trophic support for neurons and/or the production of neurotoxic mediators.

### The neurotoxicity is glia-dependent and mediated by soluble mediators

**Figure 3:**
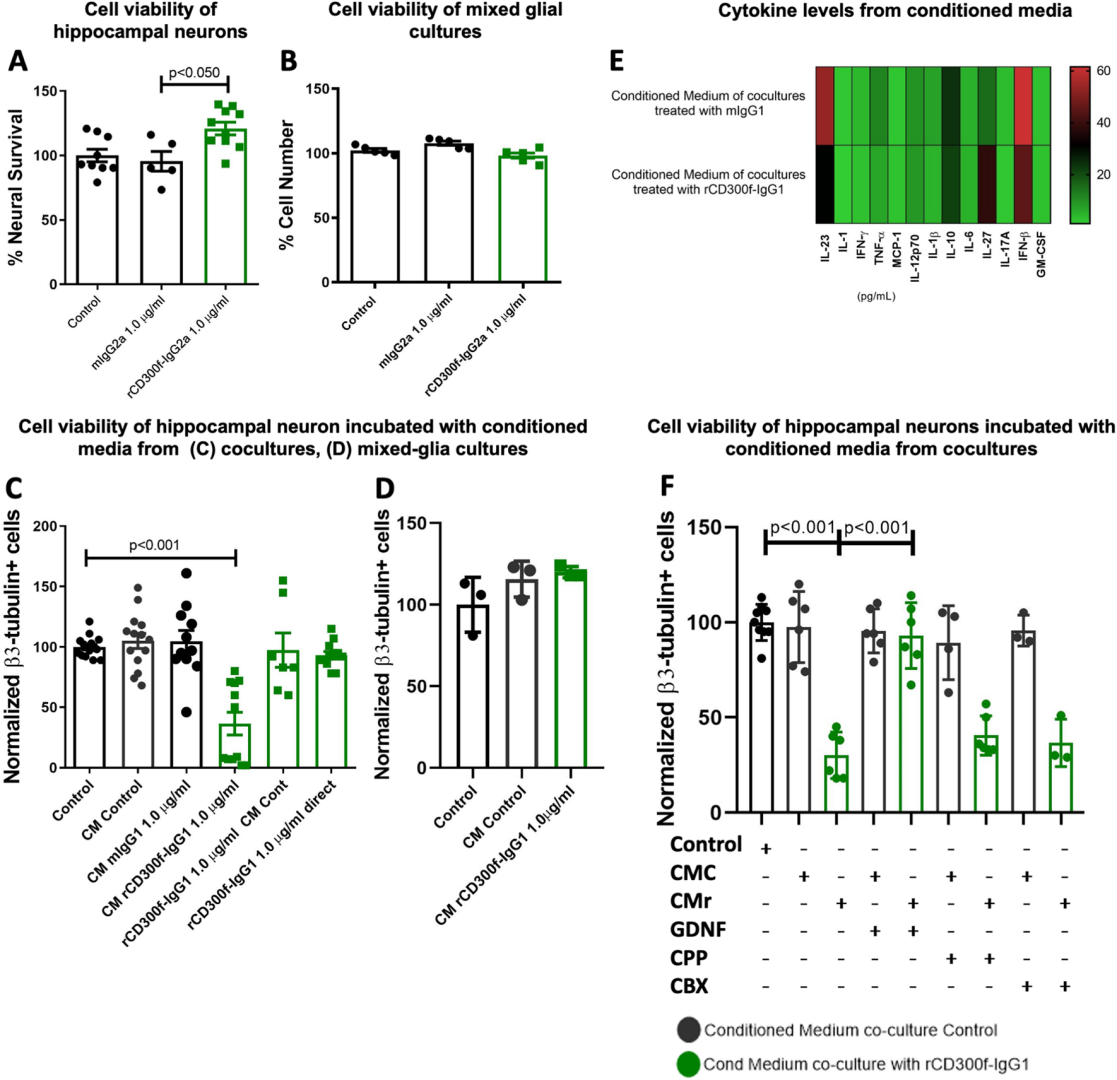
Conditioned media from high dose of CD300f-IgG-treated cocultures is neurotoxic. (**A**) Hippocampal enriched neuronal cultures were incubated with rCD300f-IgG2a or control IgG2a and neuronal viability was defined by MTT (two independent experiments were performed). (**B**) Mixed hippocampal glial cultures were incubated with rCD300f-IgG2a or control IgG2a and cellular viability was determinate by MTT (two independent experiments were performed). (**C**) Hippocampal enriched neuronal cultures were incubated with the conditioned media from cocultures treated with rCD300f-IgG1 (1.0 μg/ml) or control IgG1 (1.0 μg/ml), as additional controls we incubated neurons with control conditioned medium and fresh rCD300f-IgG1 (1.0 μg/ml), or fresh rCD300f-IgG1 (1.0 μg/ml) (three independent experiments were performed). (**D**) Hippocampal enriched neuronal cultures were incubated with mixed-glia conditioned media (one experiment is shown here where the conditioned media was neuron media, an additional experiment with glial media is shown in Suppl. Figure 3). (**E**) Cytokine levels were measured in conditioned media from glia cultures treated with rCD300f-IgG1 and from neuron-glia cocultures treated with IgG1 and rCD300f-IgG1. (**F**) Hippocampal enriched neuronal cultures were incubated with conditioned media from cocultures treated with rCD300f-IgG1 (1.0 μg/ml) or control IgG1 (1.0 μg/ml), and co-treated with glial-derived neurotrophic factor (GDNF, 1.0 ng/ml); NMDA glutamate receptor inhibitor 3-(2-Carboxypiperazin-4-yl)propyl-1-phosphonic acid (CPP, 100 μM), or GAP junction and connexin hemichannel inhibitor carbenoxolone (CBX, 100 μM) (two independent experiments were performed). Data show mean ± SEM; p corresponds to one way ANOVA followed by Tukey’s test (**A**–**F**).

To unravel if the mechanism by which rCD300f-Fc promotes neurotoxicity is cell-type specific, we incubated hippocampal enriched neuronal or mixed glial cultures with rCD300f-IgG2a or control IgG2a for 72 h and analyzed cell viability using the MTT reduction method. rCD300f-IgG2a did not affect neuronal survival in neuron-enriched cultures compared to controls, however, it induced a significative increase in MTT reduction. It is now known that in viability assays, MTT is mainly reduced by the coenzyme NAD(P)H and glycolytic enzymes of the endoplasmic reticulum and therefore, cellular MTT reduction represents a measure of the rate of glycolytic NAD(P)H production^22^. Thus, our data suggest an increased metabolic activity in neurons treated with rCD300f-IgG2a. Moreover, it opens up the possibility that soluble CD300f may be interacting with an endogenous ligand expressed in neuronal surface (Fig. 3A). Moreover, treating mixed glial cultures with rCD300f-IgG2a or control IgG2a show no decrease in cell viability (Fig. 3B). To further understand the CD300f-dependent neuronal death mechanism, we analyzed whether a soluble factor secreted by glial cells or neurons could be mediating this process. Thus, we treated hippocampal enriched neuronal cultures with conditioned media derived from rCD300f-IgG1 (1.0 μg/ml) or control IgG1 (1.0 μg/ml)-treated cocultures and counted the number of surviving neurons 72 h after. We observed a strong toxic effect of the conditioned medium harvested after CD300f inhibition, with a 64 ± 11% decrease of neuronal cell number when compared to control IgG1 (Fig. 3C). Moreover, the toxicity was not mediated by a combination of the rCD300f-IgG1 to other basal components of the conditioned media, as neuronal enriched cultures showed no toxicity when incubated with control cocultures conditioned media containing fresh rCD300f-IgG1 (1.0 μg/ml, Fig. 3C). Surprisingly, the conditioned media derived from mixed glial cultures treated with rCD300f-IgG1 promotes no toxicity in neuronal enriched cultures related to the untreated control or IgG1 conditioned media (Fig. 3D and Suppl. Figure 3). Together, these results suggest that a more complex environment that includes neurons besides glial cells and soluble factors is important for the induction of the observed neurotoxicity, highlighting the role of neuron-glia interactions in this process.

CD300f immune receptor can negatively regulate proinflammatory activation of innate immune cells, including the activation of diverse Toll-like receptors^9^. We next analyzed whether inhibiting CD300f signaling would induce a proinflammatory glial phenotype and thus neurotoxicity. Hence, cocultures were incubated with rCD300f-IgG1 (1.0 μg/ml) or control IgG1 (1.0 μg/ml) and the concentration of diverse cytokines was measured in culture media after 72 h by using the Biolegend LEGENDplexTM Mouse Inflammation Panel (13-plex). No significant alterations in IL23, IL1α, IL1β, IFNγ, IFNβ, TNFα, MCP1/CCL2, IL12p70, IL10, IL6, IL17A, IL27 and GM-CSF cytokine concentration was detected under these experimental conditions(Fig. 3E).

To further characterize the neurotoxic mechanism, we incubated enriched hippocampal neuronal cultures for 72 h with conditioned media from cocultures treated with rCD300f-IgG1 or control IgG1, and co-treated with: (i) glial-derived neurotrophic factor (GDNF, 1.0 ng/ml); (ii) NMDA glutamate receptor inhibitor 3-(2-Carboxypiperazin-4-yl) propyl-1-phosphonic acid (CPP, 100 μM), or (iii) GAP junction and connexin hemichannel inhibitor carbenoxolone (CBX, 100 μM). As expected, conditioned media derived from rCD300f-IgG1-treated cocultures promoted a 70 ± 12% or 69 ± 12% neuronal death when compared to controls (Fig. 3F). Interestingly, only GDNF was able to rescue neurotoxicity, showing no significant differences in neuronal survival compared with the one elicited by conditioned media from untreated or mIgG1-treated cocultures (Fig. 3F). These results suggest that soluble mediators such as glutamate or ATP are not involved in the neurotoxic mechanism, and thus other glial mediators such as toxic lipids or proteins could be modulating this process.

### CD300f-IgG is not neurotoxic in CD300f^−/−^ mice after a penetrating brain injury

**Figure 4:**
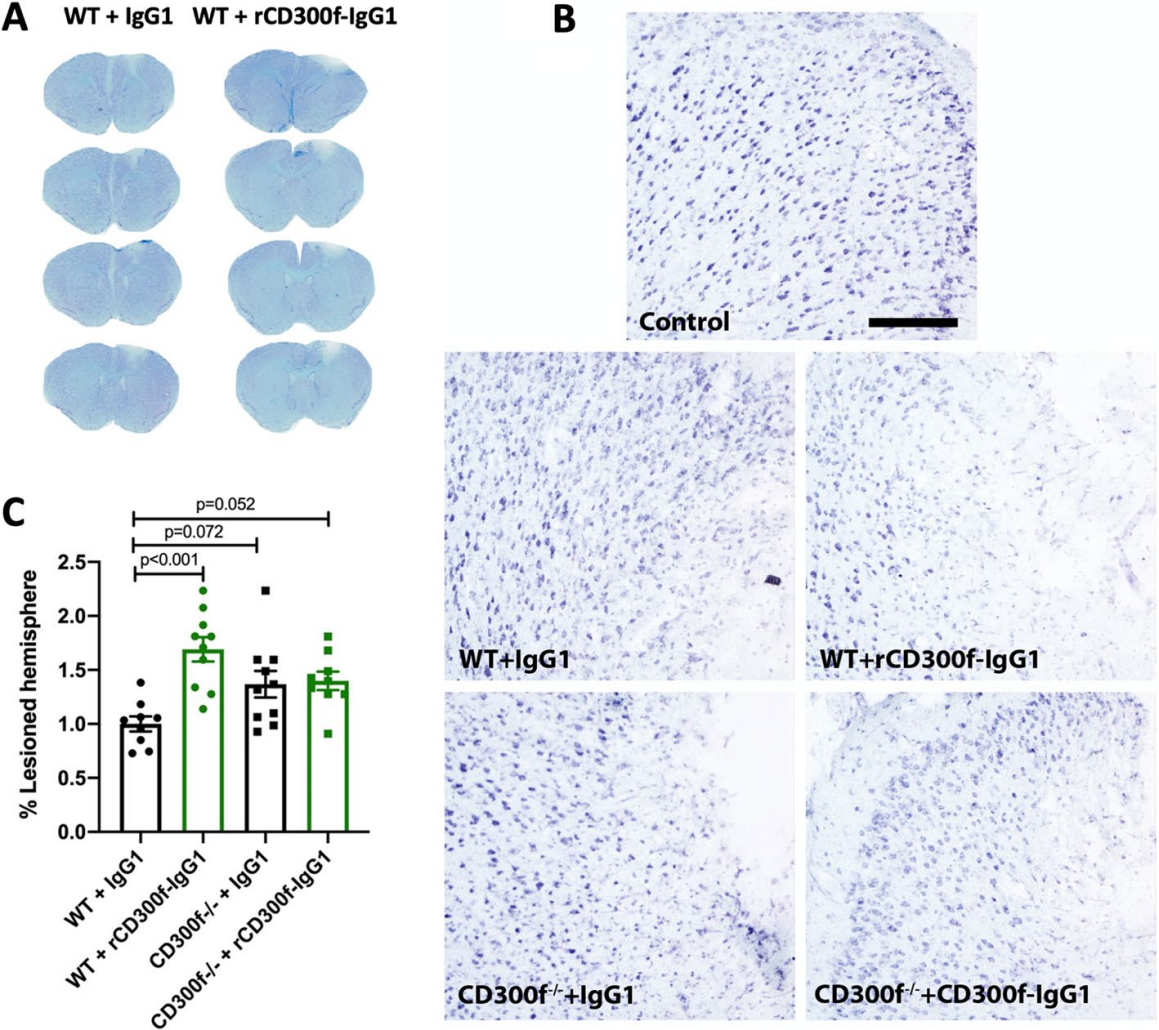
CD300f inhibition by rCD300f-Fc is specific. (**A**,**B**) Representative images of Nissl stained Sects. 72 h after WT or CD300f^−/−^ animals were intracortically injected with IgG1 or rCD300f-IgG1 (Scale bar 200 μM). (**C**) Analysis of lesion volume was performed by Nissl staining 72 h after (WT injection of IgG1 n = 9, rCD300f-IgG1 n = 10, KO injection of IgG1 n = 10 and rCD300f-IgG1 n = 9). Two independent experiments were performed in different days including all treatments in each day. Data show mean ± SEM; p corresponds to one way ANOVA followed by Tukey’s test.

Many CD300f ligands, such as phosphatidylserine, sphingomyelin or lipoproteins, are shared with other members of the CD300 family of immune receptors and with TREM2 immune receptor^23–25^. To address the question of whether the effects of rCD300f-Fc could be mediated by the inhibition of other immune receptors, CD300f^−/−^ mice brains were injected intracortically with rCD300f-Fc (30 μg/ml) or control IgG (30 μg/ml) and the % of lesioned hemisphere was evaluated 72 h after by Nissl stain (Fig. 4A,B). As expected for a specific effect, while CD300f-IgG1 injection was neurotoxic for WT animals (70 ± 4% increased lesioned hemisphere when compared to control IgG1), no significant effect was observed by CD300f-IgG1 injection in CD300f^−/−^ mice when compared to control IgG injection (Fig. 4C). Together, these data suggest that the neurotoxic effect induced by the fusion protein is indeed due to the inhibition of the endogenous CD300f receptor. Moreover, the intracortical injection of the control IgG1 or rCD300f-IgG1 into CD300f^−/−^ mice showed a strong trend towards increasing the lesion volume when compared to WT mice injected with control IgGs (40 ± 4% increased lesioned hemisphere, *p* = 0.072 or 40 ± 3% increased lesioned hemisphere, *p* = 0.052 respectively) (Fig. 4C), suggesting that the absence of CD300f is neurotoxic after a penetrating brain injury.

## Discussion

Here, we show that the CD300f immune receptor contributes to neuronal survival after a lesion by participating in glial-neuron interactions. Our data suggest that tonic microglial CD300f activation is necessary for maintaining healthy trophic interactions, and when disrupted, can trigger neurotoxic responses. In accordance, several lines of evidence suggest that tonic activation of TREM2 lipid-binding immune receptor also contributes to CNS homeostasis^1,26^. The tonic activation of CD300f and TREM2 lipid-binding immune receptors could be triggered by lipoprotein recognition, or myelin recycling structures. It still remains to be clarified which are the actual endogenous ligands under physiological conditions for these receptors.

Important lessons for understanding CD300f function may be learned from TREM2 biology, as both receptors share functions, part of their cell-type specific expression, they are putatively activated by similar ligands, and their signaling pathways are intermingled. In fact, CD300 immune receptors are the closest relatives to TREM receptors^1^. The spectrum of described ligands shared by TREM2 and CD300f include phospholipids and lipoproteins^8,27–30^. In particular, they recognize phospholipids such as phosphatidylserine and sphingomyelin that will contribute to sense and phagocytose apoptotic cells, and cellular debris such as myelin debris. TREM2 also recognizes Aβ, and though not clearly demonstrated, this could also be the case for CD300f and its closest relative CD300b, as they were found to interact with Aβ in an unbiased screening study using protein arrays^31^. In this way, these receptors may act as pathogen-associated molecular patterns (PAMP’s) or damage-associated molecular patterns (DAMP’s) receptors, enabling innate immune cells to rapidly recognize tissue damage or extracellular misfolded proteins that triggers interconnected signaling pathways contributing to microglial and barrier macrophage sensing of the surrounding events occurring in the CNS parenchyma (reviewed in^32^).

Though sharing similar phospholipid ligands, other molecules may act as co-ligands conferring specificity for different lipid-binding immune receptors. Staining cells with TREM2-Fc or CD300f-Fc fusion proteins show that many, but not all live cells, display ligands for these receptors despite abundant phospholipids ligands such as sphingomyelin exposed in the surface of all cells. Moreover, the staining of cells with CD300f-Fc show a patched profile highlighting specific cell surface domains where the ligands are exposed^11^. Taken together, these observations open the possibility that other molecules apart from the phospholipids, such as proteins, may act as co-receptors with phospholipids generating and additional level of complexity and specificity. We show here that, while the in vivo injection of rCD300f-Fc into WT brain induces an increase in lesion volume when compared to control IgGs, there is a similar lesion volume after the injection of rCD300f-Fc or control IgGs into CD300f^−/−^ brains. This denotes that while the rCD300f-Fc protein inhibits the endogenous CD300f-ligand interactions in WT brains, it does not affect the CD300f^−/−^ brain. More importantly, the lack of effects of rCD300f-Fc in CD300f^−/−^ brains evidences that, though CD300f may share lipid ligands with other lipid-binding receptors, it possesses additional ligands that confer specific activation of this receptor. The lack of in vivo effect of the rCD300f-Fc fusion protein in CD300f^−/−^ brain also ensures that the fusion protein is a specific tool for blocking CD300f-ligand interactions. Finally, the injection of control IgGs or rCD300f-Fc into CD300f^−/−^ brains showed an increased lesion volume when compared to the injection of control IgGs into WT brains, confirming that indeed CD300f signaling deficiency induces enhanced neurotoxicity in vivo.

The inhibition of microglial CD300f-induced neurotoxicity, which was dependent on the presence of both neurons and glial cells, was mediated by soluble factors, and could be reversed by GDNF treatment. Thus, inhibiting microglial CD300f signaling could lead to a proinflammatory state toxic for cocultured neurons. In this sense, it has been shown that CD300f regulates microglial immunometabolic phenotype, being necessary for boosting its metabolism in response to inflammatory stimuli^7^. CD300f^−/−^ mice showed increased depressive-like behaviors, that were potentiated by systemic and CNS inflammation induced by intraperitoneal LPS injection^7^. Depression has been shown to be induced under inflammatory conditions, and CD300f has been show to dampen inflammatory reactions in different systemic conditions^8,13,33^. Thus, CD300f^−/−^ mice could display depressive-like behaviors due to low-grade CNS inflammation induced by the release of the negative signaling brake. However, only minor inflammatory profile of the brain or isolated microglial cells were altered in CD300f^−/−^ mice^7^. Accordingly, the neurotoxicity observed after CD300f inhibition was not mediated by an increased proinflammatory cytokine release. We also showed that the underlying toxic mechanisms do not involve NMDA-dependent excitotoxicity, nor extracellular ATP-dependent toxicity. Regarding the cell-type responsible for neurotoxicity, as CD300f is only expressed in microglia and barrier macrophages in the CNS and mostly by myeloid cells in the periphery^5^, it may be exerted directly by CD300f-expressing microglia and/or recruited macrophages or neutrophils after the penetrating brain injury. However, in cocultures, the CD300f-dependent neurotoxicity seems to be dependent on microglial cells, as they are the only CD300f-expressing cells in this experimental conditions^34,35^. Alternatively, CD300f inhibition may affect microglia-dependent protective mechanisms, as has been shown in vitro and after CNS lesions, where a TREM2-dependent microglial scavenging of toxic lipids such as oxidated phosphatidylcholine occurs^36^. Based on their ligand similarities, CD300f could putatively also scavenge oxidated phosphatidylcholine after the penetrating brain injury, while the inhibition of this process would potentiate neurotoxicity. Moreover, microglia can induce an astrocyte neurotoxic phenotype, a toxicity that is maintained in astrocyte conditioned media^37^. This effect was later found to be dependent on toxic long-chain saturated free fatty acids associated to APOE and APOJ lipoproteins^38^. Astrocytes have also been shown to be capable of inducing neurotoxic responses by producing reactive oxygen and nitrogen species^39^, however these species are not stable in conditioned media and thus not responsible of the neurotoxic effect. Further studies will be needed for the identification of the toxic components involved in this process and the cell type that produce them.

Most immune receptors such as TREM1, CD200R, TREM2, and CD300f display soluble forms. A major source of soluble forms is the translation of an alternatively spliced transcript that lacks the transmembrane domain, and this is the case for both human and mouse CD300f^5,10^. However, proteolytic cleavage of surface-expressed immune receptors by matrix metalloproteases may also contribute to soluble forms production^1^. Evidence shows that the cleavage of cell surface immune receptors terminates their signaling, and reflects their activation level in vivo. Moreover, soluble forms may have their own functions, such as scavenging ligands and further restraining immune receptor activation, or even activating yet unknown receptors. For instance, soluble CD300b activates TLR4^40^. In addition, a beneficial effect has been reported after soluble TREM2 brain injection or overexpression in an Aβ pathology mice model^41^. Human longitudinal studies of Alzheimer’s disease patients show that a higher CSF soluble TREM2 levels predicts slower cognitive decline and attenuated amyloid and tau signal in positron-emission tomography^42,43^*.* This is in contrast with the neurotoxic effects of soluble CD300f injection into normal mouse and rat brain reported here, suggesting that in spite of all the similarities between these two immune receptors they may also have their particularities.

Taken together, the results presented here and previous results related to CD300f^7,10,35^, as well as the extensive work of other colleagues on CD200R1/CD200^44,45^, TREM2^1^, and Siglec^46,47^ receptors support the notion that immune receptors contribute to restoring nervous system homeostasis after a lesion or under chronic neurodegenerative conditions, and constitute key regulators of microglial phenotype. Importantly, we also show evidences that, though lipid-binding immune receptors share many lipidic ligands, there are additional conditions or co-ligands that confer different specificities.

### Supplementary Information

Supplementary Figures.

## Data Availability

All data will be available upon request to Hugo Peluffo.
