# Supplementary material for: Microglial CD300f immune receptor contributes to the maintenance of neuron viability in vitro and after a penetrating brain injury
Source: Sci Rep. 2023 Oct 5;13:16796. doi: 10.1038/s41598-023-43840-1 (PMC10556028; doi:10.1038/s41598-023-43840-1)
Supplement: Supplementary file 1 — Supplementary Figures. [file 41598_2023_43840_MOESM1_ESM.pdf]

Microglial CD300f immune receptor contributes to the maintenance of neuron viability *in vitro* and after a penetrating brain injury

Daniela Alí-Ruiz[1, 2], Nathalia Vitureira[3] and Hugo Peluffo*[1, 2, 4, 5]

[1]Neuroinflammation and Gene Therapy Lab., Institut Pasteur de Montevideo, Uruguay

[2]Dep. of Histology and Embryology, Faculty of Medicine, UdelaR, Montevideo, Uruguay

[3]Dep. of Physiology, Faculty of Medicine, UdelaR, Montevideo, Uruguay

[4]Unitat de Bioquímica i Biología Molecular, Departamento de Biomedicina, Facultat de Medicina i Ciències de la Salut, Universitat de Barcelona (UB), Barcelona, Spain

[5]Institut de Neurociències, Universitat de Barcelona (UB), Barcelona, Spain

* Corresponding author: Hugo Peluffo

email: hugo.peluffo@pasteur.edu.uy

Supplementary figure 1

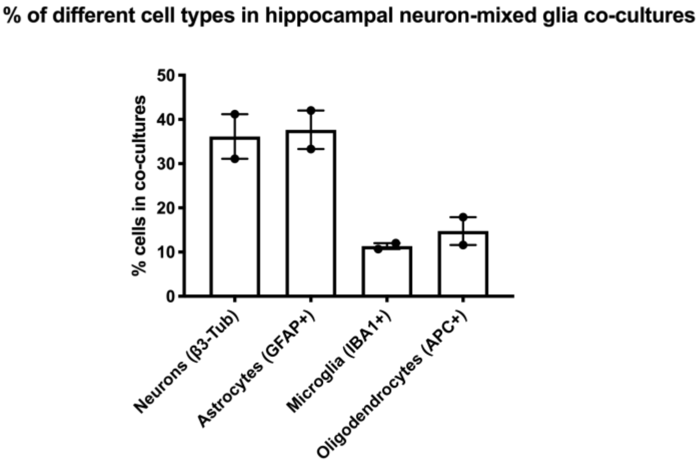

Figure 1S. The cellular composition of hippocampal neuron-glia cocultures. Immunolabeling against several cell-type specific markers. We detected 36% βIII-Tubulin positive neurons, 38% GFAP positive astrocytes, 11% IBA1 positive microglia and 15% APC positive oligodendrocytes. Total cells were stained with DAPI. Data show mean ± SEM.

Supplementary figure 2

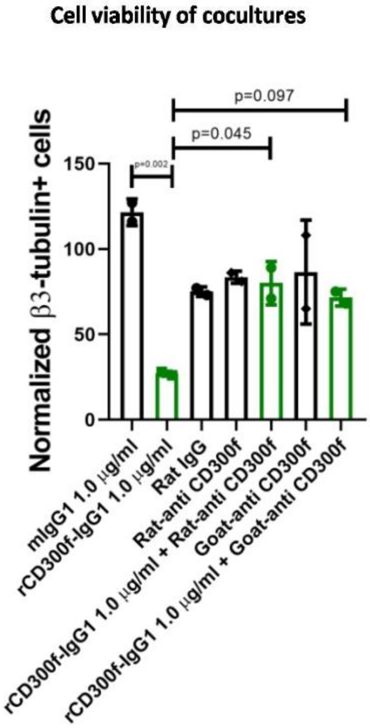

Figure 2S. The toxic effect for rCD300f-Fc was prevented by two different anti-CD300f antibodies. Cocultures were incubated with rCD300f-IgG1 (1μg/ml) or control IgG1 (1μg/ml) plus the following treatment: control IgGs or two polyclonal anti-CD300f antibodies (rat monoclonal IgG1 or goat polyclonal as indicated in the Methods section). Cultures were also incubated only with the anti-CD300f

antibodies alone. β3-Tubulin positive cell numbers were counted 72 hours after and are represented as normalized to control β3-Tubulin+cells. Data correspond to on experiment and show mean ± SEM; p corresponds to one way ANOVA followed by Tukey's test.

Supplementary figure 3

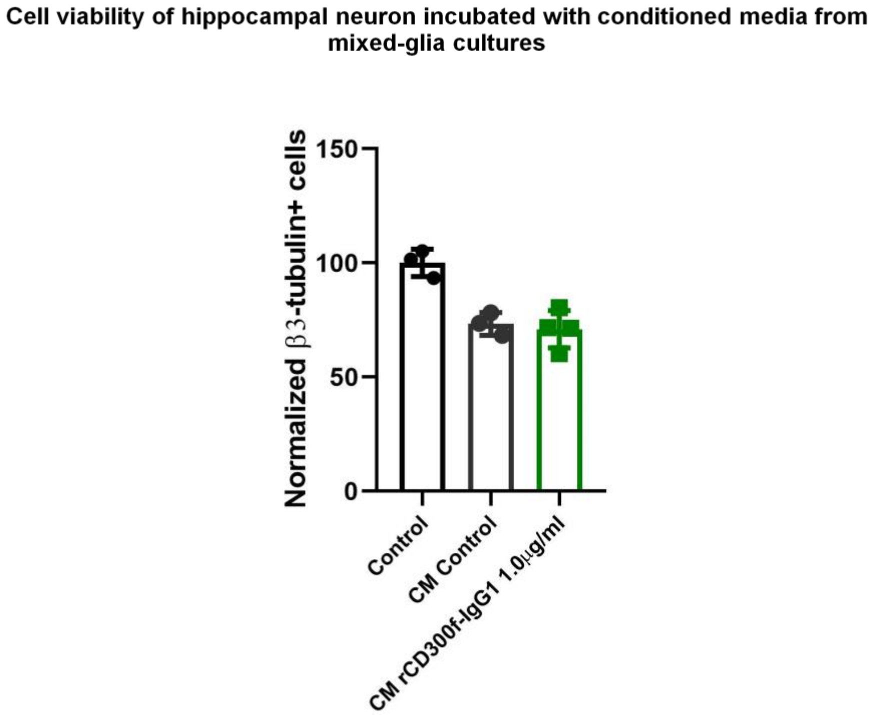

Figure 3S. Conditioned media from CD300f-IgG1 treated mixed glial culture is not neurotoxic. In this experiment, mixed glial cultures were incubated with BME-based culture media (GibCo) containing 10% fetal calf serum (GibCo), Glutamax (GibCo), 20 mM D-glucose (GibCo), HEPES (1%), pyruvate (1%) and penicillin/streptomycin (GibCo) and rCD300f-IgG1 (1.0 μg/ml) or control IgG1 (1.0 μg/ml). Then, hippocampal neuron enriched cultures were incubated with the different mixed-glia conditioned media and β3-Tubulin positive cell numbers were counted 72 hours after. Data are represented as normalized to control β3-Tubulin+cells, correspond to on experiment and show mean ± SEM; p corresponds to one way ANOVA followed by Tukey's test.
